# Genetic and Quantitative Trait Locus Analysis for Bio-Oil Compounds after Fast Pyrolysis in Maize Cobs

**DOI:** 10.1371/journal.pone.0145845

**Published:** 2016-01-08

**Authors:** Brandon Jeffrey, Najeeb Kuzhiyil, Natalia de Leon, Thomas Lübberstedt

**Affiliations:** 1 Department of Agronomy, Iowa State University, Ames, IA, United States of America; 2 Department of Mechanical Engineering, Iowa State University, Ames, IA, United States of America; 3 Department of Agronomy, University of Wisconsin, Madison, WI, United States of America; China Agricultural University, CHINA

## Abstract

Fast pyrolysis has been identified as one of the biorenewable conversion platforms that could be a part of an alternative energy future, but it has not yet received the same attention as cellulosic ethanol in the analysis of genetic inheritance within potential feedstocks such as maize. Ten bio-oil compounds were measured via pyrolysis/gas chromatography-mass spectrometry (Py/GC-MS) in maize cobs. 184 recombinant inbred lines (RILs) of the intermated B73 x Mo17 (IBM) Syn4 population were analyzed in two environments, using 1339 markers, for quantitative trait locus (QTL) mapping. QTL mapping was performed using composite interval mapping with significance thresholds established by 1000 permutations at α = 0.05. 50 QTL were found in total across those ten traits with R^2^ values ranging from 1.7 to 5.8%, indicating a complex quantitative inheritance of these traits.

## Introduction

Corn cobs and stover have received attention for use as alternative energy platforms. It is estimated that more than 280 million tons of these residues are produced each year in the United States [1, 2]. Jansen and Lübberstedt [2] reviewed some of the current and projected uses for maize cobs and concluded that dual purpose maize is feasible. While the grain fraction would be used for other purposes, cobs would serve as a bioenergy feedstock. The use of cobs and stover as bioenergy feedstocks has raised concerns due to a potentially negative impact on soil organic matter, and thus soil fertility [3, 4]. However, the fast pyrolysis platform offers a solution to this problem. Bio-char, the solid product of fast pyrolysis, can be applied to the soil in order to return carbon and minerals [5].

While there have been several quantitative trait locus (QTL) mapping experiments completed in maize in regards to ethanol conversion [6, 7], thermochemical conversion (such as fast pyrolysis) has yet to receive the same attention in this area, despite the potential to produce a wider variety of products [8]. The optimal plant ideotype for ethanol conversion likely differs from the ideotype for fast pyrolysis. Lignin has been shown to decrease the amount of ethanol produced from biomass by limiting enzymatic activity necessary to convert cellulose into sugars, and ultimately ethanol [9, 10]. Lignin’s role in bio-oil production is not as clear, as a higher lignin content increases bio-oil yield and confers a higher heating value, but also negatively affects stability [11, 12]. It is this difference in lignin contributions that will likely require separate breeding strategies for these two alternative energy platforms. In addition, there are efforts towards converting hemicelluloses into ethanol in order to increase conversion efficiency [13]. However, pyrolysis of hemicelluloses creates a greater amount of char, acid, and gas than cellulose, which do not contribute to higher bio-oil yield and quality [14, 15].

Water content for bio-oil can vary from 15–30% and heating values range from 16–19 Megajoules (MJ)/kg, while petroleum oils typically contain very little water (0.1%) and have a higher heating value of 40 MJ/kg [16]. Petroleum oils are composed almost exclusively of hydrocarbons. In contrast, bio-oil is a much more complex mixture that is composed of many different types and sizes of compounds (of which most are decomposition products of cellulose, hemicelluloses, or lignin), and its elemental composition mirrors that of the original biomass, with a much higher oxygen content than petroleum oils (35–40% versus 1%, respectively) [8]. By contrast, ethanol is a much less complex fuel, but requires engine modification for higher concentrations and is incompatible with the current infrastructure in the United States [17]. Because the various compounds in the bio-oil differ in their desirability and value, it is reasonable to suggest that bio-oil quality could be significantly improved through plant breeding. Bio-oil can also be refined or fractionated to provide high value chemicals and products, as well as bio-precursors for multiple uses (like petroleum oils, but in contrast to ethanol). Given genetic variation for individual bio-oil compounds, it is conceivable to create crop varieties to maximize yield of particular high value products.

Jeffrey *et al*. [18] demonstrated significant genetic variation for bio-oil compounds within maize cobs and stover among isogenic *brown midrib* hybrids of maize. In this study, measurements for 26 compounds were available. However, we focused on ten of those compounds for QTL analysis. Two compounds each were chosen that derive from cellulose, hemicelluloses, and the hydroxyphenyl, guaiacyl, and syringyl lignin subunits. Levoglucosan and hydroxyacetaldehyde derive from cellulose [19–21], and levoglucosan has been identified as being a potentially important economic compound due to its use in pharmaceuticals, surfactants, and polymer manufacturing as well as a possible role in a hybrid thermochemical/biological processing environment by being hydrolyzed to glucose, which can then be fermented to ethanol [16, 22]. Hydroxyacetaldehyde has interest in the food flavoring industry as it is a component in “liquid smoke.” Acetic acid derives from hemicelluloses [14], and is of interest because it can be extracted for use as a chemical. However, the low pH of bio-oil (to which acetic acid contributes) can cause usage and storage issues [16]. Hydroxyacetone has been identified as a product of hemicellulose [23] and cellulose pyrolysis [21, 24]. Phenolic compounds produced through pyrolysis can be used in food flavoring agents and resins [16]. In addition, pyrolysis/gas chromatography-mass spectrometry (Py/GC-MS) has received attention as an analysis tool for identifying cell wall fractions, especially in differentiating lignins [25–27]. We have chosen phenol and 4-methylphenol for hydroxyphenyl, vanillin and 2-methoxyphenol for guaiacyl, acetosyringone and 2,6-dimethoxyphenol for syringyl lignin subunits. 2,6-Dimethoxyphenol, acetosyringone, and vanillin were all measured as part of a study that assessed variation among nine maize inbred lines, plus one *brown midrib3* mutant, for pyrolysis of neutral detergent fiber [25]. Phenol, 2,6-dimethoxyphenol, 2-methoxyphenol, and vanillin were evaluated to determine cell wall lignin and polysaccharide differences amongst stover samples for ten commercial maize hybrids grown in Italy [26].

The intermated B73 x Mo17 (IBM) Syn4 population is an advanced intercross lines (AILs) population that was developed in order to provide increased QTL resolution. The population was developed through four generations of random mating after the creation of the F_2_ generation and has an overall map distance almost four times greater than previously used recombinant inbred lines (RILs) [28].

Our objectives were to (1) determine whether there is significant genetic variation for 10 pyrolysis compounds in the IBM Syn4 population and to calculate phenotypic correlations among these compounds, (2) identify QTL for those 10 compounds, and (3) compare our results with previous QTL studies on maize cell wall traits and discuss implications for breeding dual purpose maize.

## Materials and Methods

### Plant Materials

Plant materials used in this study have been previously described by Lorenz *et* al. [6] and Jansen *et al*. [29]. Briefly, Lorenz *et* al. [6] analyzed genetic variation and correlations for yield, digestibility, and cell wall composition traits and found 24 QTL across five cell wall traits for per se and testcross families in the IBM Syn4 population. Jansen *et al*. [29] analyzed variation and correlations for grain and cob yield traits (cob length, weight, volume, density, diameter, pith diameter, wooden part thickness) and found 57 QTL across eight traits.

Field trials were performed in 2006 and 2007 in Madison (43° 3'19.73"N, 89°31'56.42"W) and Arlington (43°18'13.57"N 89°23'16.10"W), WI and were planted in a randomized complete block design with two replications per location (i.e. one replication is one complete block). B73 (parent), Mo17 (parent), and 206 recombinant inbred lines (RILs) of the IBM Syn4 per se populations were planted at a density of 79,040 plants per hectare in single row plots that were 6.08m long and 0.76m apart. Trials were planted in Plano silt loam soil on May 21, 2006 in Madison and June 2, 2006 in Arlington. After most of the plots at a location reached physiological maturity, all plots at that location were harvested. In 2006, all ears were harvested by hand 125 days after planting in Madison and 114 days after planting in Arlington. Cobs were then shelled and dried in a forced-air dryer for one week at 55°C. Cobs were ground in a hammer mill to pass a 1mm screen. Ground cobs were further ground in a ball mill (Spex SamplePrep 200 Geno/Grinder, Metuchen, NJ, United States) to reduce particle size. Each parent (B73 and Mo17) was planted in two plots per block. Phenotypic data for the parents were not used in QTL analysis, but included for comparison to each other and the RILs.

In this study, we used cob materials from 2006: one field replication from Madison and two field replications from Arlington, WI. 184 RILs from the IBM Syn4 per se population were phenotyped for 26 bio-oil compounds through pyrolysis/gas chromatography-mass spectrometry (Py/GC-MS). Field permits were not required, as trials were performed on University of Wisconsin-Madison field plots dedicated to experimentation. Field trials did not involve endangered or protected species.

### Py/GC-MS

The method and instruments used are the same described by Jeffrey *et al*. [18]. Briefly, each 500 μg ground cob sample was pyrolyzed at 500°C using a double shot pyrolyzer. Helium gas carried pyrolysis vapors directly into a gas chromatograph (GC), which used a 14% cyanopropyl polysiloxane capillary column to separate the compounds. A single quadropole mass spectrometer (MS) operating at a mass to charge ratio (m/z) of 40 to 650 was used to detect compounds. Peak areas were acquired from the total ion current (TIC) chromatogram using proprietary software (Agilent Technologies, Santa Clara, CA, United States). Areas from each of the 26 compounds were divided by the total area in the TIC chromatogram (and multiplied by 100) to obtain an area % value for each compound.

### Statistical Analyses

Least square means (lsmeans) were calculated for each RIL over all 3 environments in SAS PROC GLM (SAS Institute, 2004), using RIL and environment (three environments: the two replications from Arlington were considered as distinct environments) as fixed effects. Least square means for each RIL were used as phenotypic data for correlation and QTL analysis. Phenotypic correlations were calculated as Pearson product-moment coefficients using SAS PROC CORR. Coefficients of variation (CV) were calculated by dividing square root of the mean square error by mean (multiplied by 100). Phenotypic data for B73 and Mo17 were compared against each other using SAS PROC GLM, with genotype and environment as fixed effects.

Heritabilities were calculated according to Holland *et al*. [30] on an entry mean basis using SAS PROC MIXED by fitting lines, locations, lines x locations (G x E), and field replications as random effects.

### QTL Analysis

Genotypic data for 1339 markers were obtained for 184 RILs from MaizeGDB.org (IBM302 map provided by the Maize Mapping Project, http://www.maizegdb.org/qtl-data.php, verified 3-7-2013). This map provides a length of 6242.7centimorgans (cM), which conveys an average distance between markers of 4.66cM. This map does not convey cM in the traditional definition, thus we will refer to positions on the map in IBM centimorgans (IcM) [31].

Composite interval mapping (CIM) was performed using WinQTL Cartographer version 2.5 [32]. Ten cofactors were identified using forward and backward regression (Zmap model 6) with a 10cM window size, 1.0cM walk speed, and a 0.10 probability for inclusion/exclusion. An empirical threshold value for determining significant QTL for all traits was determined using 1000 permutations at α = 0.05. For each trait, all significant QTL were fitted in a multiple interval mapping (MIM) model to determine whether those QTL remained significant. In addition, additive model effects, individual QTL R^2^ effects, and sum of R^2^ effects over all QTL of a trait were calculated using MIM.

## Results

### Trait Means, Variances, and Heritabilities

Means, standard errors, heritabilities, and other summary statistics for the ten compounds are shown in Table 1. Line and environment effects were significant (p < 0.001) for all ten compounds. For each compound, the RIL with the minimum area % value for that compound was lower than the mean of both B73 and Mo17. Also for each compound, the RIL with the maximum area % value for that compound was higher than the mean of both B73 and Mo17. The mean of neither B73 nor Mo17 ranked overly high or low within individual compounds, as the highest rank achieved (out of 186) was 42 and the lowest was 164. B73 had a significantly (p < 0.05) higher mean than Mo17 for levoglucosan, hydroxyacetaldehyde, vanillin, and acetosyringone. Mo17 had a significantly (p < 0.05) higher mean than B73 for acetic acid and phenol, while the other four compounds showed no evidence to reject the hypothesis that B73 and Mo17 have equal means. Coefficients of variation for the data ranged from 9.31% (acetic acid) to 26.71% (levoglucosan). Heritabilities (entry mean basis) ranged from 0.24 for 4-methylphenol to 0.84 for acetosyringone (Table 1). The majority of heritabilities (7 out of 10) were high and had values that met or exceeded 0.62.

**Table 1:** Compound Summary Statistics.

Trait	#	N	Mean	RMSE	CV	B73	Mo17	Min	Max	H^2^
Levoglucosan	1	541	1.15	0.31	26.71	1.44	0.88	0.53	2.13	0.69
Hydroxyacetaldehyde	2	541	0.77	0.10	12.55	0.86	0.76	0.50	0.91	0.33
Acetic acid	3	541	8.58	0.80	9.31	8.26	9.29	5.90	10.42	0.48
Hydroxyacetone	4	541	4.60	0.46	9.89	4.37	4.91	3.50	6.07	0.67
Phenol	5	541	0.73	0.08	11.56	0.71	0.81	0.46	1.00	0.62
4-Methylphenol	6	541	0.267	0.03	12.15	0.26	0.29	0.18	0.33	0.24
Vanillin	7	541	0.59	0.07	12.42	0.66	0.54	0.32	0.86	0.66
2-Methoxyphenol	8	541	1.20	0.12	9.75	1.31	1.21	0.82	1.73	0.79
Acetosyringone	9	539	0.10	0.02	19.78	0.14	0.08	0.05	0.22	0.84
2,6-Dimethoxyphenol	10	541	0.77	0.10	13.11	0.74	0.66	0.34	1.24	0.83

Values are given, by compound, for mean, square root of the mean square error (RMSE), coefficient of variation (CV), B73 and Mo17 lsmeans, minimum line lsmean, maximum line lsmean, and entry mean heritability (H^2^).

### Trait Correlations

Overall, loose trait correlations were found (Table 2), with the closest positive correlation being 0.80 (between vanillin and 2-methoxyphenol) and the most negative correlation being -0.56 (between levoglucosan and hydroxyacetone). Compounds derived from the same cell wall component had generally closer trait correlations than compounds derived from different cell wall components. The loosest of these correlations was between cellulose derived compounds levoglucosan and hydroxyacetaldehyde at 0.39. The correlation between hemicellulose derived compounds acetic acid and hydroxyacetone was 0.71. The correlation between hydroxyphenyl derived compounds phenol and 4-methylphenol was 0.73. The closest of these correlations (and amongst all correlations) was between guaiacyl derived compounds vanillin and 2-methoxyphenol at 0.80. The correlations between syringyl derived compounds acetosyringone and 2,6-dimethoxyphenol was 0.56. The only compounds that had a correlation greater than 0.60 with other compounds not derived from the same cell wall component were between both of the guaiacyl derived compounds and both of the syringyl derived compounds: vanillin had a correlation of 0.60 with both of the syringyl derived compounds and 2-methoxyphenol had a correlation of 0.66 with acetosyringone and 0.67 with 2,6-dimethpxyphenol.

**Table 2:** Phenotypic Correlations Among Compounds.

Trait	1	2	3	4	5	6	7	8	9	10
1		0.39**	0.30**	-0.56**	-0.39**	-0.14**	0.39**	0.11**	0.27**	0.01
2			0.21**	0.23**	-0.21**	-0.06	-0.03	-0.14**	-0.05	-0.14**
3				0.71**	0.27**	0.35**	-0.45**	-0.31**	-0.31**	-0.27**
4					0.45**	0.34**	-0.30**	-0.04	-0.11*	0.04
5						0.73**	0.28**	0.45**	0.14**	0.42**
6							0.06	0.18**	-0.02	0.17**
7								0.80**	0.60**	0.60**
8									0.66**	0.67**
9										0.56**
10										

(1) levoglucosan, (2) hydroxyacetaldehyde, (3) acetic acid, (4) hydroxyacetone, (5) phenol, (6) 4-methylphenol, (7) vanillin, (8) 2-methoxyphenol, (9) acetosyringone, (10) 2,6-dimethoxyphenol.

* indicates a p-value < 0.05

** indicates a p-value < 0.01

### QTL Analysis

**Fig 1:**
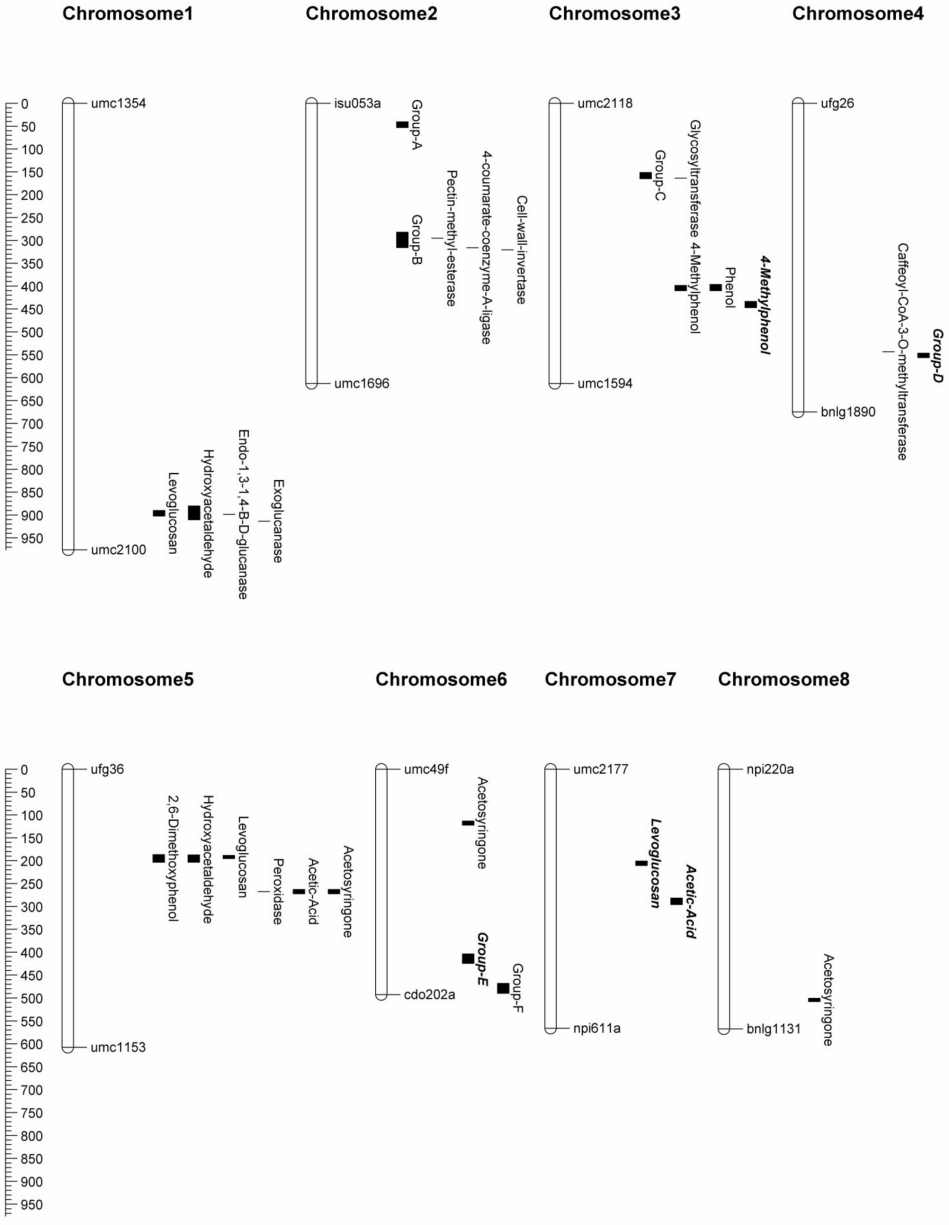
Chromosome map. Chromosomes 9 and 10 are not pictured, as we found no QTL on these chromosomes. Chromosome number is indicated at the top of each chromosome. QTL positions and candidate gene positions from Truntzler *et al*. [39] are indicated on the right side of each chromosome. QTL indicated as groups contained 4 or more individual QTL in the same region. Individual QTL contained within groups are shown in Fig 2. QTL names in ***bold italics*** indicate a higher area % value for the B73 allele, with regularly formatted text indicating a higher area % for the Mo17 allele.

**Fig 2:**
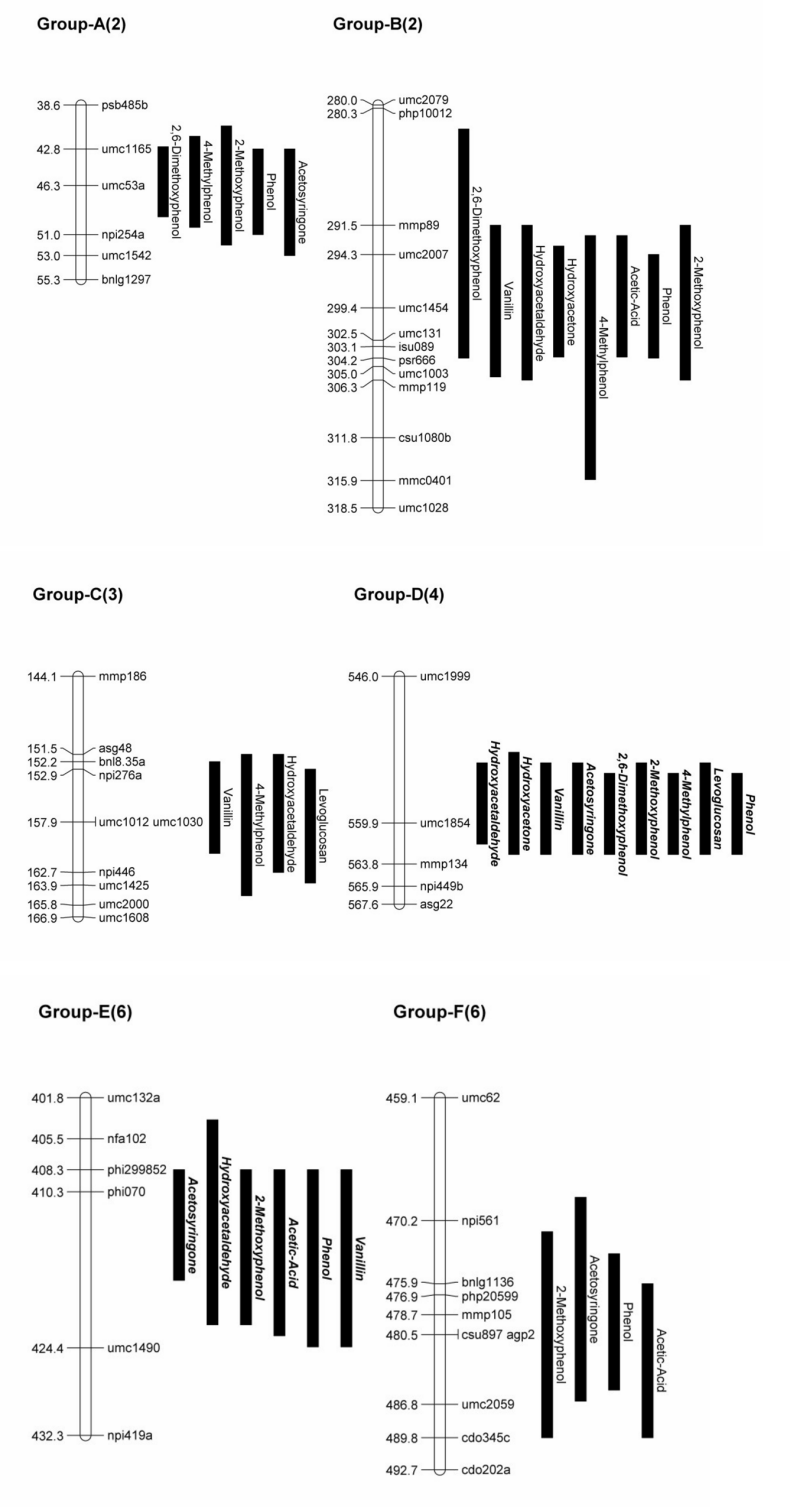
Expanded chromosome map. Close up view of QTL Groups A through G. Group name from Fig 1 is shown at the top of the chromosome with the chromosome number indicated in parentheses. QTL names in ***bold italics*** indicate a higher area % value for the B73 allele, with regularly formatted text indicating a higher area % for the Mo17 allele.

QTL analysis resulted in the identification of 50 QTL across eight maize chromosomes, as no QTL for these traits were found on chromosomes 9 or 10 (Table 3, Figs 1 and 2). All 50 QTL remained significant when fitting them in a MIM model, although the position of the QTL sometimes shifted slightly. The amount of phenotypic variation explained by individual QTL ranged from 3.0% to 7.4% (with an average of 4.1%) under the CIM model and ranged from 1.7% to 5.8% (with an average of 3.6%) under the MIM model. The average 1-LOD support interval spanned 12.31IcM.

**Table 3:** QTL Positions and Effects.

Chromosome	Position (IcM)	Bin	Marker	1-LOD Interval	LOD	R^2^	α	R^2^_MIM_	α_MIM_
**Levoglucosan**									
1	892.8	1.1	umc1111	890.3–902.2	3.38	3.8	-0.13	4.4	-0.13
3	157.9	3.03–3.04	umc1012	152.9–163.7	3.56	4.3	-0.13	3.6	-0.12
4	560.9	4.08–4.09	umc1854	554.2–562.9	2.65	3.0	0.11	2.1	0.09
5	192.3	5.02–5.03	bnl7.56	188.6–195.3	3.65	4.2	-0.13	3.4	-0.12
7	206.5	7.02	umc2092	200.9–210.5	2.84	3.2	0.12	3.9	0.13
**Hydroxyacetaldehyde**									
1	892.8	1.1	umc1111	880.4–910.2	3.18	3.7	-0.08	3.2	-0.07
2	298.3	2.04	umc1454	291.5–306.3	2.89	3.5	-0.08	3.9	-0.07
3	156.9	3.03–3.04	umc1012	151.5–162.7	2.51	3.1	-0.07	3.2	-0.07
4	557.1	4.08–4.09	umc1854	554.2–561.9	3.33	3.8	0.08	3.2	0.07
5	192.3	5.02–5.03	bnl7.56	187.6–203.4	2.99	3.5	-0.07	2.7	-0.07
6	412.3	6.06	phi070	403.8–422.3	2.92	4.0	0.08	3.6	0.07
**Acetic Acid**									
2	299.4	2.04	umc1454	292.5–315.8	3.26	3.8	-0.87	5.7	-1.07
5	267.3	5.03	bnl4.36	263.2–272.3	2.84	3.4	-0.81	2.6	-0.69
6	414.3	6.06	phi070	408.3–423.3	3.02	4.6	0.94	4.0	0.95
6	484.5	6.07	umc2059	475.9–489.8	3.03	4.0	-0.90	2.3	-0.87
7	288.9	7.02–7.03	npi394	281.6–295.9	4.40	7.4	1.32	2.4	0.70
**Hydroxyacetone**									
2	299.3	2.04	umc1454	293.5–304.1	4.26	5.4	-0.55	5.4	-0.53
4	557.1	4.08–4.09	umc1854	553.2–562.9	3.05	3.6	0.46	3.9	0.45
**Phenol**									
2	46.3	2.01–2.02	umc53a	42.8–51.0	3.42	4.1	-0.08	3.1	-0.07
2	299.4	2.04	umc1454	294.3–304.2	3.79	4.4	-0.08	5.0	-0.09
3	406.5	3.06	mmp5	396.6–409.9	3.33	4.3	-0.08	1.7	-0.05
4	560.9	4.08–4.09	umc1854	555.2–562.9	2.99	3.5	0.07	3.6	0.07
6	414.3	6.06	phi070	408.3–424.3	2.79	4.3	0.08	3.6	0.08
6	477.9	6.07	mmp105	473.2–485.5	3.72	4.2	-0.08	2.4	-0.07
**4-Methylphenol**									
2	45.8	2.01–2.02	umc53a	41.6–50.3	3.59	4.6	-0.04	2.7	-0.02
2	299.4	2.04	umc1454	292.5–315.8	3.34	4.0	-0.03	4.1	-0.03
3	156.9	3.03–3.04	umc1012	151.5–164.9	2.77	3.4	-0.03	2.8	-0.02
3	406.5	3.06	mmp5	398.6–409.9	3.12	4.2	-0.03	2.6	-0.03
3	438.9	3.06	php15033	433.7–447.1	2.56	3.2	0.03	1.8	0.02
4	560.9	4.08–4.09	umc1854	555.2–562.9	3.23	3.9	0.03	2.9	0.02
**Vanillin**									
2	297.3	2.04	umc1454	291.5–306.0	3.64	4.6	-0.07	3.5	-0.05
3	155.9	3.03–3.04	umc1012	152.2–160.9	3.50	4.5	-0.07	4.2	-0.06
4	557.1	4.08–4.09	umc1854	554.2–562.9	3.63	4.2	0.06	3.7	0.06
6	414.3	6.06	phi070	408.3–424.3	2.66	3.9	0.06	3.5	0.06
**2-Methoxyphenol**									
2	46.3	2.01–2.02	umc53a	40.6–52.0	2.82	3.5	-0.11	4.3	-0.12
2	300.4	2.04	umc1454	291.5–306.3	2.60	3.1	-0.11	4.1	-0.13
4	560.9	4.08–4.09	umc1854	554.2–562.9	3.32	3.7	0.12	4.4	0.13
6	413.3	6.06	phi070	408.3–422.3	3.09	4.6	0.13	3.9	0.13
6	477.9	6.07	mmp105	471.2–489.8	2.80	3.2	-0.11	2.0	-0.10
**Acetosyringone**									
2	47.3	2.01–2.02	umc53a	42.8–53.0	4.56	5.3	-0.01	5.4	-0.01
4	558.1	4.08–4.09	umc1854	554.2–562.9	3.82	4.3	0.01	5.3	0.01
5	267.3	5.03	bnl4.36	263.2–272.3	4.09	4.5	-0.01	4.0	-0.01
6	118.4	6.01–6.02	mmp117	113.3–122.1	3.48	3.8	-0.01	3.2	-0.01
6	411.3	6.06	phi070	408.3–418.3	4.67	5.8	0.01	4.6	0.01
6	477.9	6.07	mmp105	468.1–486.5	2.74	3.0	-0.01	1.8	-0.01
8	502.9	8.07–8.08	umc1673	500.7–507.9	2.87	3.1	-0.01	3.9	-0.01
**2,6-Dimethoxyphenol**									
2	44.8	2.01–2.02	umc53a	42.6–49.3	4.55	5.7	-0.13	4.1	-0.08
2	297.3	2.04	umc1454	282.3–304.2	3.12	3.6	-0.08	4.9	-0.09
4	560.9	4.08–4.09	umc1854	555.2–562.9	5.65	6.2	0.11	5.8	0.10
5	192.3	5.02–5.03	bnl7.56	186.8–203.4	3.79	4.2	-0.08	2.6	-0.07

The number of QTL for each compound, chromosome number, chromosome position, bin, closest marker, Logarithm of the odds (LOD) score, 1-LOD support interval, phenotypic variance explained under the CIM model (R^2^), additive effect for the B73 allele under the CIM model (α), phenotypic variance explained under the MIM model (R^2^_MIM_), and additive effect for the B73 allele under the MIM model (α_MIM_) are shown. Data reported in Table 3 reflects values for lsmeans of combined environment.

The 50 QTL were spread across 15 different (greater than 10IcM separating QTL positions) chromosome regions, with 6 regions containing 4 or more QTL (Fig 1). The first region was located on chromosome 2 (with QTL positions spanning 44.8IcM to 47.3cM) with QTL for five compounds: phenol, 4-methylphenol, 2-methoxyphenol, acetosyringone, and 2,6-dimethoxyphenol. The region with the most QTL (9) was located on chromosome 4 around position 560.9IcM, with acetic acid being the only compound without a QTL in this region. Chromosome 2 around position 298.7IcM contained QTL for eight compounds, with only levoglucosan and acetosyringone not having QTL in this region. QTL for six compounds were found around position 413.1IcM on chromosome 6, with QTL for hydroxyacetaldehyde, acetic acid, phenol, vanillin, 2-methoxyphenol, and acetosyringone. The two remaining regions contained four QTL each and were located on chromosome 6 around position 484.4IcM and on chromosome 3 around 158.7IcM. The region on chromosome 3 had QTL for levoglucosan, hydroxyacetaldehyde, 4-methylphenol, and vanillin, while the region on chromosome 6 shared QTL for acetic acid, phenol, 2-methoxyphenol, and acetosyringone.

Five QTL were found for levoglucosan that accounted for 18.5% of the phenotypic variation (17.4% under the MIM model): on chromosome 1 at position 892.8IcM, chromosome 3 at 157.9IcM, chromosome 4 at 560.9IcM, chromosome 5 at 192.3IcM, and chromosome 7 at 206.5IcM. Four of these QTL mapped to the same region as the other cellulose derived compound (hydroxyacetaldehyde) and were located on chromosomes 1, 3, 4, and 5, respectively. The other two QTL found for hydroxyacetaldehyde were on chromosome 2 at 298.3IcM and on chromosome 6 at 412.3IcM. Together these six QTL accounted for 21.6% of the phenotypic variation (19.8% under the MIM model).

Five QTL were found for acetic acid that accounted for 23.2% of the phenotypic variation (17.0% under the MIM model): on chromosomes 2 (299.4IcM), 5 (267.3IcM), 6 (414.3 and 484.5IcM), and 7 (288.9IcM). One of those QTL locations was also found for the other hemicellulose derived compound (hydroxyacetone) on chromosome 2. The second QTL for hydroxyacetone was identified on chromosome 4 at 557.1IcM, and the two QTL together accounted for 9.0% of the phenotypic variation (9.3% under the MIM model).

Six QTL were found for phenol that accounted for 24.8% of the phenotypic variation (19.4% under the MIM model): chromosome 2 at 46.3IcM and 299.4IcM, chromosome 3 at 406.5IcM, chromosome 4 at 560.9IcM, and chromosome 6 at 414.3IcM and 477.9IcM. For the other hydroxyphenyl derived compound, 4-methylphenol, four QTL mapped to the same region as those for phenol: both QTL on chromosome 2, chromosome 3 at 407.0IcM, and on chromosome 4. The other two QTL found for 4-methylphenol were found on chromosome 3 at 156.9IcM and 438.9IcM. The six QTL for 4-methylphenol accounted for 23.3% of the phenotypic variation (16.9% under the MIM model).

Five QTL were found for 2-methoxyphenol that accounted for 18.1% of the phenotypic variation (18.7% under the MIM model): chromosome 2 at 46.3IcM and 300.4IcM, chromosome 4 at 560.9IcM, and chromosome 6 at 413.3IcM and 477.9IcM. Three of those QTL were also found in similar regions in the other guaiacyl derived compound, vanillin: chromosome 2 at 297.3IcM, chromosome 4 at 557.1IcM, and chromosome 6 at 414.3IcM. One other QTL was found for vanillin on chromosome 3 at 155.9IcM and the four QTL accounted for 17.2% of the phenotypic variation (14.9% under the MIM model).

The most QTL we found for a compound (7) were for acetosyringone and accounted for 29.8% of the phenotypic variance (28.2% under the MIM model). These occurred on chromosome 2 at position 47.3IcM, chromosome 4 at 558.1IcM, chromosome 5 at 267.3IcM, chromosome 6 at 118.4IcM and 411.3IcM and 477.9IcM, and chromosome 8 at 502.9IcM. Two of these QTL were found in similar regions for the other syringyl derived compound, 2,6-dimethoxyphenol: chromosome 2 at 44.8IcM and chromosome 4 at 560.9IcM. Two other QTL were found for 2,6-dimethoxyphenol: chromosome 2 at 297.3IcM and chromosome 5 at 192.3IcM. These four QTL accounted for 19.7% of the phenotypic variation (17.4% under the MIM model).

## Discussion

Due to exclusively finding QTL with minor genetic effects, we conclude that compounds resulting from pyrolysis can be considered as quantitative traits in the IBM Syn4 population. Jansen *et al*. [29] found an average of four QTL per cob yield or cob quality trait with an average explained phenotypic variance of 6.5% in the IBM Syn4 population, while Lorenz *et al*. [6] found an average of 2.4 QTL per cell wall trait with an average explained phenotypic variance of 9.4%. We found an average of five QTL per trait with an average explained phenotypic variance of 4.1%. By assuming absence of dominance and gene interaction effects, we can estimate the amount of heritability explained by the QTL we found, by dividing total R^2^ by heritability for each trait, R^2^/h^2^. Explained heritability ranged from 13.5% to 95.5% under the CIM model with an average of 39.8%, and from 13.9% to 69.3% with an average of 33.6% under the MIM model. On average, 60.2% of heritability went unexplained.

Heritability is measured as a proportion of variance explained by genetic effects in relation to overall phenotypic variance. Sources of genetic variance include additive, dominance, and epistatic effects. Phenotypic variance includes all genetic variance plus environmental variance, genotype x environment interaction variance, and twice the covariance between genotype and environment. Most analyses, including ours, ignore epistasis because it is too complex to model and estimate for a large number of genes or QTL. However, this can lead to an overestimation of heritability, and therefore, an underestimation of explained heritability [33]. In addition, statistical power of QTL detection and a large number of QTL with very small effects will cause QTL to be missed [33, 34]. Because we found no QTL with explained phenotypic variance greater than 7.4%, it is likely that bio-oil compounds are affected by a large number of QTL with small effects.

We found the closest correlations among compounds derived from the same cell wall polymers. Consistent with these findings, QTL co-located for these compounds. Different compounds with close correlations, such as the guaiacyl and syringyl derived compounds, also shared several QTL. Of the six distinct chromosomal regions that contained QTL for guaiacyl derived compounds, five also had QTL for a syringyl derived compound. While this could be due to either pleiotropy or close linkage of QTL, it is notable that we found 50 QTL in only 15 different regions. In consequence, we identified a limited number of regions with co-locating QTL.

For lignin related compounds, there are six regions with co-locating QTL. On chromosome 2, five QTL for lignin derived compounds were located between 44.8 and 47.3IcM. All five of these QTL had higher area % values for the Mo17 allele. Five additional QTL for lignin derived compounds were found on chromosome 2 between 297.3 and 300.4IcM. The favorable allele for maximizing lignin derived compounds was contributed by Mo17 for all five. QTL for hydroxyacetaldehyde, hydroxyacetone, and acetic acid were found in this region as well, with the trait increasing allele also coming from Mo17. Increased acetic acid content is likely to be undesirable as the low pH of bio-oil can cause usage and storage issues [16]. If the eight QTL in this region are due to pleiotropy, one would have to weigh the costs and benefits of these contrasting compounds to determine whether the B73 or Mo17 allele is preferable. In case of closely linked QTL, it would be possible to separate these QTL spanning a 3IcM region [35].

While no QTL mapping studies have been previously performed for pyrolysis related traits, it is reasonable to compare our results to QTL studies for cell wall traits, ethanol conversion, or forage and digestibility traits, since all of these traits relate to cell wall composition.

Barrière *et al*. [36] found 80 QTL in total for cell wall digestibility and composition traits in a population of 242 RILs derived from the cross of F838 and F286. Five different regions from their study overlap with QTL found in our study. They found QTL for esterified ferulic acids and vanillin on chromosome 2 near marker bnlg1018, which maps closely (294.2IcM) to QTL that we found for eight compounds (297.3IcM–300.4IcM), including the guaiacyl derived vanillin and 2-methoxyphenol. In this region, we also found QTL for the hemicelluloses derived compounds acetic acid and hydroxyacetone. Ferulic and diferulic acids form ester and ether bonds that link lignins to arabinoxylans, which are found in hemicelluloses [37, 38]. In particular, ferulic acids are linked through an ether bond with coniferyl alcohol [39], which gives rise to guaiacyl units. Based on a candidate gene list compiled by Truntzler *et al*. [40], there are three candidate genes whose closest marker is located near this region. 4-coumarate; coenzyme A ligase (4CL) was identified as a likely candidate gene and its closest marker is positioned close to 316IcM in our map. Cell wall invertase and pectin methyl esterase were identified as “medium evidence” candidate genes, whose closest markers were positioned in our map at 320.7IcM and 295.1IcM, respectively.

On chromosome 3, Barrière *et al*. [36] found QTL for *p*-coumaric acid (PCA), syringaldehyde, and diferulic acids. The closest marker to these QTL was umc1425, located at 165IcM in our map, which is close to four QTL identified in our study (155.9IcM-157.9IcM. We found a QTL for vanillin in this region, which agrees with the finding of a QTL for diferulic acids, due to their relationship with guaiacyl units. A “medium evidence” candidate gene [40], a putative glycosyltransferase (quasimodo), was located near a marker positioned at 163.5IcM on our chromosome 3 map. QTL for acid detergent lignin/neutral detergent fiber (ADL/NDF), vanillin, and syringaldehyde were found on chromosome 7 by Barrière *et al*. [36], with the closest marker being bnlg1808 (which is positioned at 286.3IcM on our map). We found a QTL for acetic acid in this region (288.9IcM), which has been shown to be a major product of hemicellulose pyrolysis and a minor product of cellulose pyrolysis [14, 21]. While we did not find any QTL for lignin derived compounds in this region, a connection can still be made, since a higher portion of cellulose and/or hemicelluloses might lead to a different ADL/NDF ratio. The final region in common between these studies occurred on chromosome 8, with Barrière *et al*. [36] finding QTL for in vitro neutral detergent fiber digestibility (IVNDFD), ADL/NDF, klason lignin/NDF, PCA, and syringaldehyde. The closest marker (bnlg1065) to these QTL was a proximal flanking marker to all of the QTL, whose distance ranged from 9-31cM (on their map) distal of the marker. In our map, this marker is positioned at 460.8IcM. We found a QTL for acetosyringone close to this region (502.9IcM), which agrees with QTL for all five of the traits found by Barrière *et al*. [36].

We found QTL in several other regions where Truntzler *et al*. [40] identified candidate genes. On chromosome 1, we found QTL for the cellulose derived compounds levoglucosan and hydroxyacetaldehyde at 892.8IcM. Truntzler *et al*. [40] identified an endo-1,3–1,4-β-D-glucanase and an exoglucanase as medium evidence candidate genes in this region (near bnlg1268 and bnlg1671a, respectively), which map near our QTL (898.7IcM and 913.4IcM, respectively). The region with the most (nine) QTL in our study was located on chromosome 4 that is close to a high evidence candidate gene, caffeoyl-CoA 3-O-methyltransferase 4 (CCoAOMT), which is found around 550-551IcM in our map. CCoAOMT is thought to be involved in guaiacyl unit synthesis [41]. Another high evidence candidate gene, a peroxidase located near marker umc2296 (267.5IcM) on chromosome 5, mapped very closely to QTL for acetic acid and acetosyringone (267.3IcM). Peroxidases are involved in the polymerization of lignin [41], which could explain why we found a QTL for acetosyringone in this region.

Truntzler *et al*. [40] performed a QTL meta-analysis across 11 different studies for four digestibility traits and 22 cell wall composition traits in maize. The resulting composite map contained several meta-QTL in common with QTL from our study. A digestibility trait meta-QTL located on chromosome 2 was located in the same bin (2.04) as QTL for seven traits, including five lignin derived compounds. Meta-QTL for digestibility and cell wall traits on chromosome 3 in bins 3.04 and 3.06 are in common with all seven of our QTL located on chromosome 3, of which five are lignin derived compounds.

Lorenzana *et al*. [7] used an IBM Syn4 testcross population to map cell wall composition and ethanol traits in maize stover: klason lignin, glucose, xylose, arabinose, uronic acids, galactose, mannose, *p*-coumarate esters, ferulate esters, and glucose release. There are a number of regions found in this study for cell wall and ethanol traits that overlapped with regions in our study. Lorenzana *et al*. [7] found a QTL for PCA on chromosome 2 at 302.5IcM. In that same area, we found QTL for eight of our traits with the most interesting being2,6-dimethoxyphenol (297.3IcM), as this compound derives from syringyl (S) lignin units after the pyrolysis process, and PCA can be an indicator of S units as it is esterified to side chains of syringyl alcohol [41, 42]. Lorenzana *et al*. [7] also found QTL for PCA on chromosomes 5 (195.6IcM) and 7 (212.0IcM), where we found QTL for several traits. We found a QTL for the syringyl derived compound 2,6-dimethoxyphenol on chromosome 5 at 192.3IcM. QTL for glucose, mannose, and galactose were found around 204-211IcM by Lorenzana *et al*. [7] that were near a QTL for the cellulose derived compound levoglucosan (206.5IcM). They also found a QTL for arabinose on chromosome 7 located at 298.9IcM that mapped near our QTL for the hemicellulose derived compound acetic acid (288.9IcM). The final region in common is a QTL for ferulic acid on chromosome 6 at 475.9IcM [7]. We found QTL for 2-methoxyphenol and acetic acid in this region (477.9IcM and 484.5IcM, respectively) that are consistent with this finding, as ferulic acids can link hemicelluloses to guaiacyl lignin units.

Finding these QTL across studies, for similar traits, suggests that these chromosomal regions are promising candidates for further research. In addition, common QTL between these traits provides evidence that cell wall composition prior to pyrolysis is closely tied to bio-oil composition. From a breeding perspective, this could be beneficial as less expensive and higher throughput methods to determine cell wall composition (e.g. based on near infrared reflectance spectroscopy), as compared to Py/GC-MS, are available. Because all of these studies found areas where QTL for multiple cell wall related traits co-located, it is highly likely that separate breeding programs will be required to produce maize for cellulosic ethanol conversion and maize for bio-oil conversion. Common QTL between our study and Lorenzana *et al*. [7], would in most cases require opposite alleles (B73 vs. Mo17) to be selected for lignin and hemicellulose content. For example, on chromosome 2 around 300IcM, we found QTL for five lignin derived compounds. The Mo17 allele increased area % for each of these compounds. A breeding program to select for bio-oil quality would select for the Mo17 allele, while a breeding program for cellulosic ethanol conversion would select for the B73 allele.

We found genetic variation for 10 bio-oil compounds in the IBM Syn4 population, and exclusively minor QTL, each of which explained a small amount of phenotypic variance. This information, taken together with relatively high (> 0.62) heritabilities for seven compounds, suggests that favorable maize varieties for improved bio-oil composition can be developed. Due to finding exclusively minor QTL, we expect that genomic selection, rather than marker assisted selection, would be the best strategy for a breeding program to improve maize for bio-oil conversion. By using all available markers, genome wide selection can capture more genetic variation compared to marker-assisted selection [43], and, therefore, maximize response to selection [44]. For biparental populations (which are common maize breeding populations), as few as 100 markers are sufficient for predicting breeding values when using genomic selection [45].
